# Thermal Runaway Temperature Prediction of Lithium‐Ion Battery Under Extreme High‐Temperature Shock Using Experimental and Virtual Data

**DOI:** 10.1002/advs.202511359

**Published:** 2025-09-24

**Authors:** Xiaoyu Li, Shen Zhao, Xinyu Wei, Zhentao Shen, Jun Tian, Yibo Zhang, Yanli Zhu

**Affiliations:** ^1^ School of Mechanical Engineering Hebei University of Technology Tianjin 300130 China; ^2^ China North Vehicle Research Institute Beijing 100072 China; ^3^ School of Mechatronical Engineering Beijing Institute of Technology Beijing 100081 China; ^4^ State Key Laboratory of Explosion Science and Safety Protection Beijing Institute of Technology Beijing 100081 China

**Keywords:** deep learning, high‐temperature shock, lithium‐ion battery, state of charge, temperature prediction, thermal runaway model

## Abstract

The trend toward increasing energy density in lithium‐ion batteries (LIBs) makes thermal safety more critical. However, traditional adiabatic calorimetry fails to simulate flame exposure in real fire incidents. Herein, a framework of the thermal runaway (TR) temperature prediction of LIBs is proposed using experimental and virtual data for extreme high‐temperature shock conditions. Specifically, high‐temperature shock wave‐induced TR tests are conducted on LiNi_0.5_Co_0.2_Mn_0.3_O_2_ (NCM523) and LiFePO_4_ (LFP) batteries to compare combustion behavior and TR characteristics, while obtaining realistic temperature data. A 3D conjugate heat transfer and TR coupling model is established, and characteristic temperature parameters of TR under this condition are extracted for comparison and validation of accuracy with experimental data. The simulation model further enriches TR data under different states of charge (SOC) and distances from the heat source. The virtual data generated by the simulation model compensates for insufficient TR experimental data, enabling the establishment of a data‐driven prediction model. Three different deep learning models are compared to predict the trend of TR temperature variations under different heat source distances and SOC conditions. The results indicate that the proposed framework, which combines experimental and virtual data, achieves high‐fidelity, fast‐response TR temperature predictions. The optimal framework maintains a mean absolute percentage error (MAPE) below 5% across all studied conditions.

## Graphical abstract

An integrated framework predicts lithium‐ion batteries (LIB) thermal runaway (TR) under extreme high‐temperature shock. By combining experimental data with the multiphysics‐generated virtual data, a hybrid deep learning approach is developed to accurately forecast temperature evolution for unseen scenarios. This method also quantifies how state‐of‐charge (SOC) and thermal shock intensity govern TR severity, enabling proactive safety strategies for next‐generation energy storage systems.

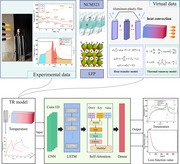

## 1 Introduction

As the global energy structure changes, electric vehicles playing an important role in decarbonizing the transport sector and have become a mainstream development in the field of transportation.^[^ ^1^, ^2^ ^]^ It is foreseeable that a large portion of the traditional vehicles worldwide will be replaced by intelligent electric vehicles. LIBs are widely used in electric vehicles and energy storage systems due to their long cycle life and high specific energy.^[^ ^3^, ^4^ ^]^ However, with the energy density of LIBs continuing to increase, the safety problems such as fires and explosions caused by thermal runaway (TR) have emerged more prominently. These concerns greatly limit broader application and development of LIBs, making safety a priority.^[^ ^5^, ^6^ ^]^

Once TR occurs, the battery temperature rises rapidly, triggering an internal exothermic chain reaction that leads to overheating.^[^ ^7^ ^]^ Abuse is the most important cause of TR in power batteries, and the abuse of LIBs includes mechanical abuse,^[^ ^8^, ^9^ ^]^ electrical abuse^[^ ^10^, ^11^ ^]^ and thermal abuse. To guarantee safety, the battery is mainly tested around these abnormal operations, including overcharge test, over‐discharge test, short‐circuit test, drop test, oven heating test, extrusion test. and pinprick test, etc.^[^ ^12^, ^13^ ^]^ Standard safety test methods under thermal abuse conditions are accelerating rate calorimeter (ARC)^[^ ^14^ ^]^ tests and oven tests.^[^ ^15^ ^]^ The ARC evaluates the initial reaction temperature, TR temperature, destructive effects, and reaction history by testing the time‐temperature‐pressure data of chemical reactions of substances under adiabatic conditions.^[^ ^16^ ^]^ The oven test simulates the TR characteristics of the battery under different external high temperatures,^[^ ^17^ ^]^ which well reflect the real accident in a safe and efficient way.^[^ ^18^ ^]^

When the temperature of the battery rises abnormally, chemical reactions occur sequentially creating a chain reaction. The battery TR is driven by the chemical crossover between the cathode and anode electrode without severe internal short circuit and voltage drop, resulting in the reaction between lithiated graphite and electrolyte.^[^ ^19^ ^]^ The positive electrode starts to decompose when battery temperature reaches to ≈200 °C. Once the ceramic coating of the separator collapsed, a large‐scale internal short circuit will bring about the burning of the electrolyte.^[^ ^20^ ^]^ Subsequently, the heat spread will cause spontaneous combustion of the entire battery pack.^[^ ^21^, ^22^ ^]^

LIBs are inevitably exposed to high temperature in field applications. Overheating is the most direct cause of TR, thus a series of thermal abuse tests have been carried out. Feng et al.^[^ ^23^ ^]^ used the extended volume‐accelerating rate calorimetry (EV‐ARC) evaluated the TR features of large capacity prismatic LIBs and found that the temperature differences inside the battery rises to the maximum when TR occurs. Mohamad et al.^[^ ^24^ ^]^ used ARC and oven characterized TR of LIBs under adiabatic and non‐adiabatic conditions, respectively. For a series of TR experiments, TR models have been established for various cases. Hatchard et al.^[^ ^25^ ^]^ first established the 1D TR model based on kinetic model for oven test of LIBs. The model considered the decomposition reaction of the solid electrolyte interface (SEI) layer, cathode, and anode. Kim et al.^[^ ^26^ ^]^ extended the 1D model of Hatchard to three dimensions, and established a 3D TR model for cylindrical LIBs for the first time. Jin et al.^[^ ^27^ ^]^ built a 3D model based on the thermal abuse tests and found that the smaller heating area that has higher heating power density can trigger TR quicker for the same heating power. Kong et al.^[^ ^28^ ^]^ developed a numerical model by coupling conjugate heat transfer with computational fluid dynamics to capture the battery temperature and internal pressure evolution under thermal abuse, venting, and subsequent combustion of 18 650 LIBs. The SOCs make a significant difference to the energy stored in LIBs and TR characteristics. Lower SOCs improves thermal stability, while higher SOCs increases self‐heating rate exponentially.^[^ ^29^ ^]^ Yan et al.^[^ ^30^ ^]^ investigated the thermal stability of LiCoO2/graphite pouch batteries under overcharging conditions and found that thermal decomposition reactions would occur at a lower temperature as the SOCs increased. He et al.^[^ ^31^ ^]^ established a 3D TR model for LiNi_0.5_Co_0.2_Mn_0.3_O_2_ (NCM523) batteries and found that the reaction onset temperature roughly increased and the enthalpy of reaction also increased with the growth of SOCs. Stefan et al.^[^ ^32^ ^]^ found that the quantity of stored Li‐ions determined the effects and strength of the TR, thus both lower SOCs and SOH minimized the severity of TR. However, the multiphysics model is complicated and computationally expensive to solve. Using multiphysics models to generate high‐quality virtual data to train data‐driven models can well compensate for this deficiency.

Data‐driven techniques are capable of automatically extracting nonlinear features and modeling complex relationships from a large amount of historical data, thus quickly capturing the complex features of TR and its evolutionary trends. Ouyang et al.^[^ ^33^ ^]^ proposed a fuzzy system and multi‐task convolutional neural network (CNN) and multiple long short‐term memory (LSTM) method to predict TR propagation multiple steps ahead. Ding et al.^[^ ^34^ ^]^ proposed a meta TR forecasting neural network using high‐dimensional thermal images as well as low‐dimensional temperature and voltage data to capture more representative thermal distributions, which predicted TR temperatures in a limited way despite limited data. Masalkovaitė et al.^[^ ^35^ ^]^ utilized a transfer learning approach to accurately estimate the change in heat output during TR using only injection mass measurements and cell metadata. Wang et al.^[^ ^36^ ^]^ explored a temperature excavation method that reveals reaction kinetic preferences hidden in minimal experiments, enables the first universal battery TR model across chemistries, formats, and working conditions. Obtaining TR data only from tests is costly and produces a single set of data, whereas the numerical simulation model could expand the dataset by adjusting the input parameters and boundary conditions, saving the cost of the tests and compensating for the lack of experimental data. Goswami et al.^[^ ^37^, ^38^ ^]^ developed a novel framework for machine learning combined with multiphysics modeling, employing a combination of graph neural network (GNN)‐LSTM to capture the temporal and spatial temperature variations generated by the multiphysics model. Jeong et al.^[^ ^39^ ^]^ proposed a multiphysics‐informed deep operator network with encoders to predict TR under various thermal abuse conditions, offering a fast and accurate TR prediction surrogate model. LIBs are subject to a series of thermal abuse tests in Underwriters Laboratories (UL) certification, Certification Bodies' Scheme (CB) certification, and CHINA QUALITY CERTIFICATION CENTRE (CQC) certification. Currently, the thermal abuse tests are mainly centered around the oven test, and the ambient temperature is usually set below 200 °C.^[^ ^40^, ^41^, ^42^ ^]^ Standardized testing typically focuses on internal failure mechanisms and fails to simulate fire and explosion incidents involving other vehicle components catching fire, battery module cascading thermal events, or high‐temperature industrial equipment malfunctioning. However, few reports exist on testing batteries under such extreme high‐temperature shock conditions,^[^ ^43^ ^]^ with insufficient relevant test data. Hence, the combination of virtual data with experimental data for temperature prediction is imperative.

Therefore, the prediction framework of TR temperature under extreme high‐temperature shock is attempted to be established for the first time. Considering two factors with significant effects, SOCs and distance from the heat source, the TR of NCM523 and LiFePO_4_ (LFP) batteries is triggered by a high‐temperature shock wave. Extreme high‐temperature shock tests are conducted to obtain real data, then a 3D coupled model of conjugate heat transfer and TR is developed for the study of batteries combustion behaviors and TR characteristics. The proposed coupled model provides sufficient virtual data to compensate for the lack of TR experimental data, which lays the foundation for building a highly accurate data‐driven prediction model. A neural network based on self‐attention mechanism of CNN combined with LSTM, bidirectional LSTM or gated recurrent unit (GRU) is established to achieve efficient and accurate prediction of TR temperature under different SOCs and different distances from the heat source.

## 2 Results and Discussion

In this section, the multiphysics model and the data‐driven models are employed to analyze and predict the temperature evolution for battery TR behavior under different abuse conditions. The multiphysics model is verified through the various abuse experiments at first. Based on the finite element model, the virtual data are extracted under different TR‐triggered conditions for making up the insufficient experiment data. Three deep learning models with different intermediate neural networks are proposed, which are applied to compare and predict the TR temperatures based on the real and virtual data.

### 2.1 TR and Burning Behavior

**Figure 1:**
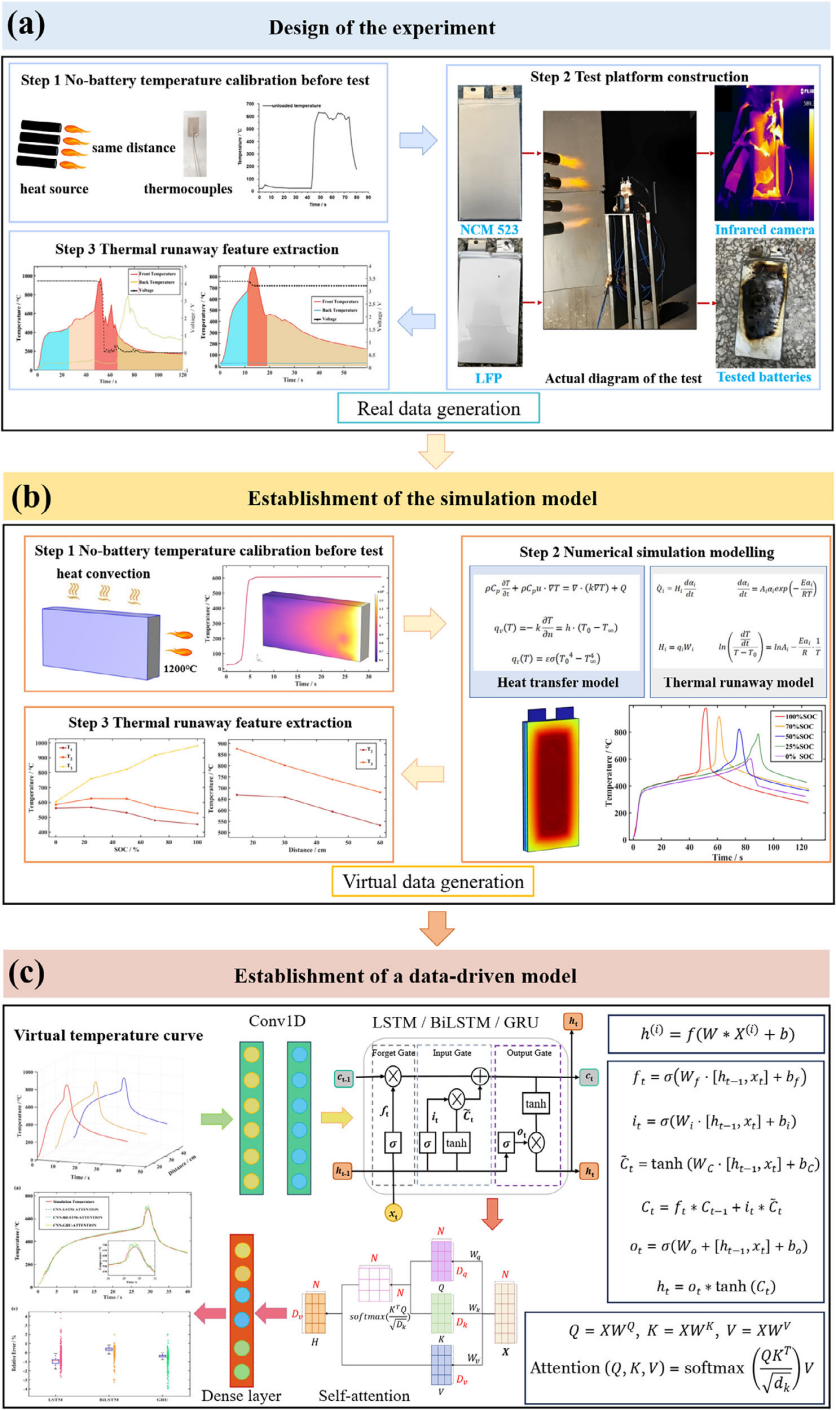
Process diagram and overall schematic of the test and modeling of this study. a) The experimental design. b) The simulation model design. c) The data‐driven model.

The single‐sided extreme high‐temperature heating test is different from the oven test in that the battery is placed in a diabatic environment. The primary difference is that the initial temperature of the external heat source in this test is extreme high, thus the rate of temperature rise is considerably higher. The tests displayed the TR behaviors of NCM523 and LFP batteries when exposed to flame shock. Different batteries showed similar combustion stages and temperature trends. Based on the characteristics of the flame in combustion combined with the temperature profile, the combustion stages are classified as 1) heat accumulation, 2) local TR initiation, 3) ignition, 4) stable combustion, 5) layer shedding, and 6) flame weakening. The combustion stages of the two types of batteries are shown in **Figure** **1**, with the first column on the left showing a sample of batteries with different cathode materials, and on the right side are images of batteries with different combustion stages.

**Figure 2:**
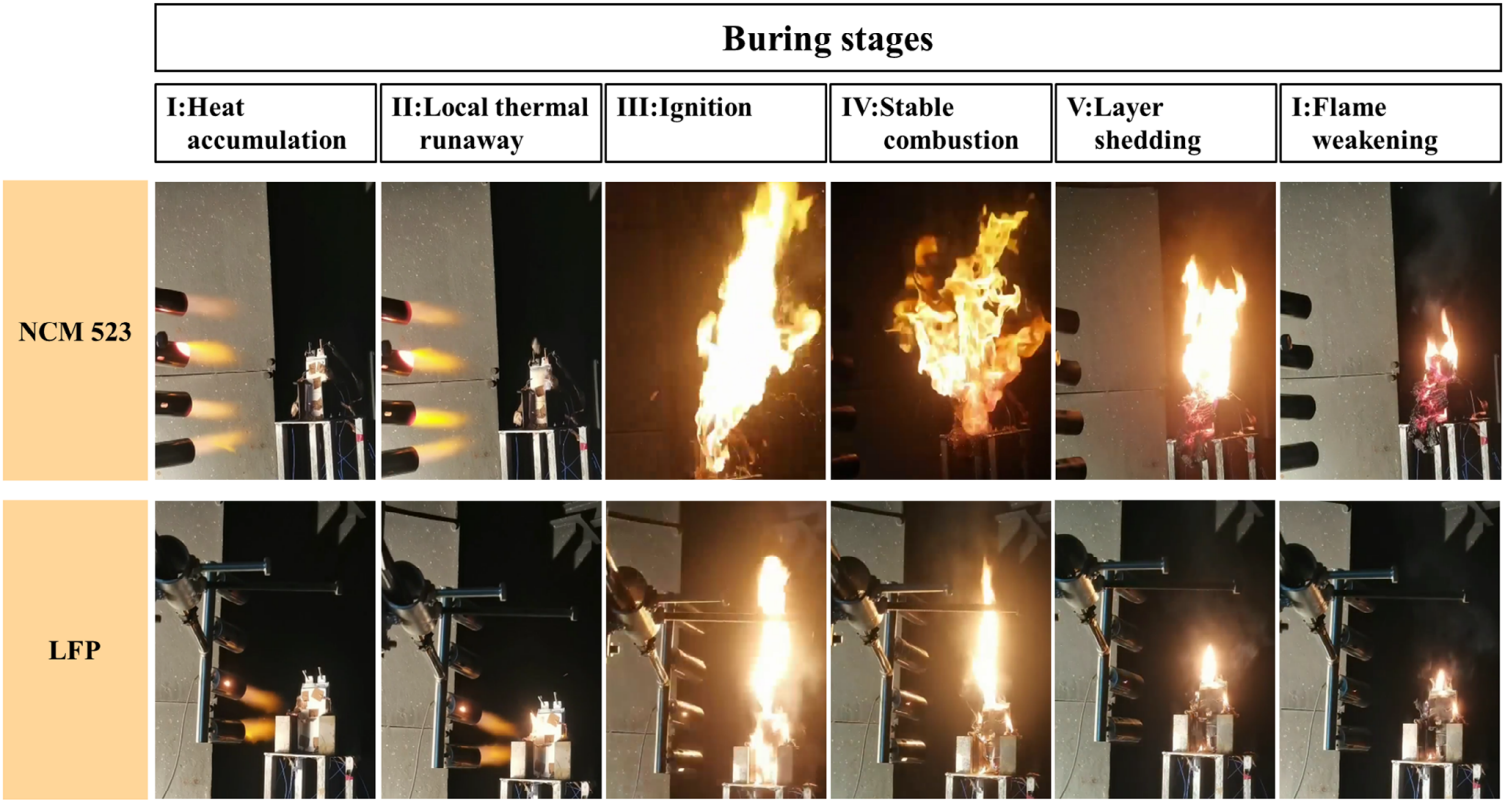
Burning stages of NCM523 and LFP.

As the external heat source is activated, the front side of the battery is exposed to the flame. In Stage I, heat rapidly accumulated in the aluminum‐plastic film shell. As the heat is transferred to the internal core, the temperature difference between the front temperature and the high temperature of the external flame is also reduced, the temperature rise rate of the front side gradually slows down. Once the temperature rise rate ascended again, indicating that the battery began to generate heat itself, local TR occurred (stage II). In this stage, the core heat generated is regarded as the dominant role leading to the temperature continually rise. Meanwhile, the TR areas are rapidly expanded until the battery starting to fire from tabs. Turned off the external heat source when the battery ignited, subsequently entered the stage III. At this time, the reaction of the positive electrode with the electrolyte and the decomposition of the electrolyte generates a considerable amount of heat. Therefore, the temperature rise rate is surged to peak, the flame size and the surface temperature reached their maximum. The voltage failed to drop to 0 V, indicating that TR occur not in all cells simultaneously, but in succession. Then the fire with the TR spread to the back of the battery. At this time, the temperature of the back side is still maintained below 100 °C, proving that the aluminum‐plastic film shell has effectively prevented the heat conduction. In stage IV, the battery combust steadily for a period of time and the flame is slightly smaller than stage III. The burned portion of the battery began to fall off from front side (stage V), and shortly after the front side fell off, battery is detected the voltage sharply drop to ≈0 V. Since the thermocouples are attached to the surface of the battery, the temperature of the battery fails to be further detected after the front is detached. However, the TR of the battery has already entered its final stage and the flames diminish gradually at this time, thus it can meet the requirements for monitoring and warning TR and has no effect on the reliability of the test. The burned battery appeared red in the center and burned to black charcoal on the outside. There is a long overlap between stage IV and V because the burning and shedding of the battery occur one after the other. As the flame burned close to the back of the battery, fewer materials could get decomposition reactions, as a result the flame kept diminishing until extinguished (stage VI). For LFP battery, the combustion stages are broadly similar to the NCM523 battery. However, the obviously difference is that LFP battery without through stage V, only the insulation shell shows relatively severe carbonization. Hence, the mass loss of LFP battery after combustion is only 0.7 g, with a mass loss rate of 0.15%, which is greatly less than that of NCM523 battery (34.3%). Notably, the LFP battery voltage only decreased slightly at the beginning of the combustion phase and stabilized at ≈3.2 V. The flame‐retardant design of this LFP battery gives it superior thermal stability. The temperatures and voltages of the two batteries are shown in **Figure** **2**.

**Figure 3:**
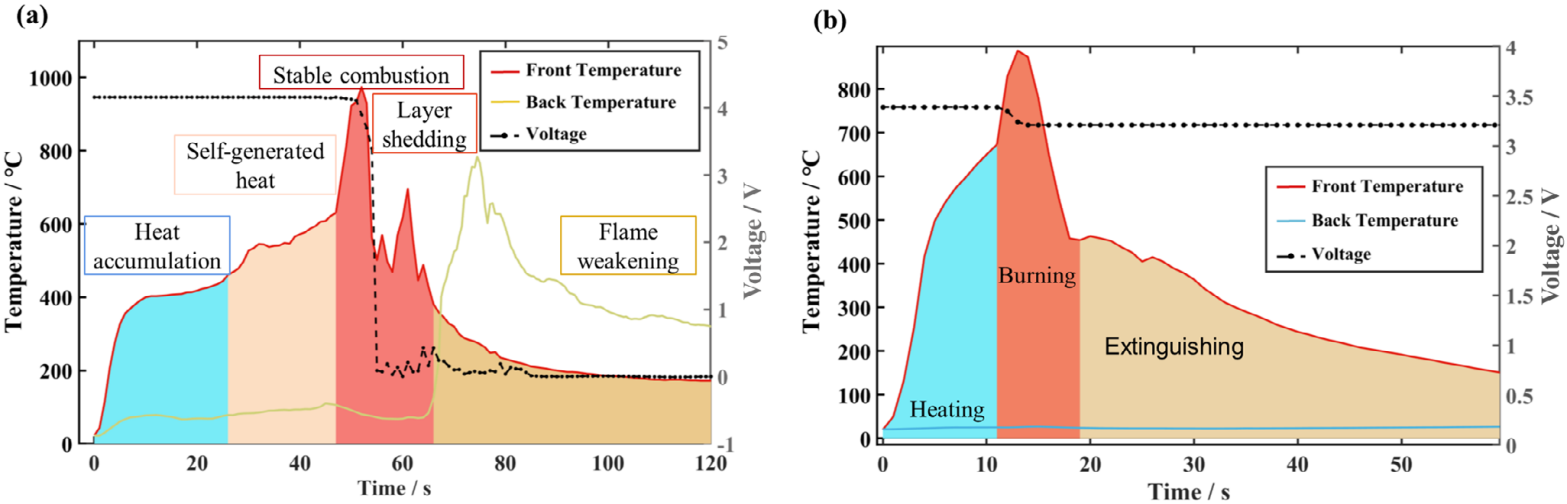
TR behaviors of the tested batteries. a) The temperature and voltage of NCM523. b) The temperature and voltage of LFP.

Comparing the combustion behavior of different materials, the LFP battery exhibits a higher onset temperature of TR and the lower peak temperature. The thermal stability advantage likely attributed to the olivine structure of LFP with strong covalent P‐O bonds. This structure inhibiting the oxygen release and requires higher decomposition enthalpy than layered oxides. Consequently, the total energy from initial exothermic reactions (primarily from anode decomposition) is insufficient to propagate complete cathode decomposition once external heating ceases, leading to self‐extinguishing behavior. In many cases, the duration of the stage IV for LFP batteries is very short. Under the high‐temperature shock conditions of this open‐environment study, LFP is considered unlikely to have undergone complete TR, as evidenced by the back surface temperature, post‐combustion morphological changes, and mass loss rate. Specifically, the maximum temperature on the back of the battery is only 43 °C, as shown in Figure 2b. After combustion, only the front shows significant swelling, as shown in **Figure** **3**a. The post‐combustion mass is 468.89 g, with a mass loss rate of only 0.31%. Moreover, there are no obvious mechanical detachment observed in LFP battery tests. Previous studies suggested that the heat release rate (HRR) of TR in an open environment may be only one‐ninth that in a closed environment.^[^ ^44^ ^]^ Furthermore, in our continuous flame test, the combustion of H_2_ emitted from the heat source consumed surrounding O_2_. This likely fails to provide a sustained, oxygen‐enriched environment necessary for complete combustion of battery components. The combination of this inherent material stability and external testing conditions resulted in the observed TR termination This confirms that LFP batteries have better thermal stability than NCM523. Once the NCM523 battery catches fire, the collapse of the layered to spinel phase releases a large amount of oxygen, causing the fire to intensify immediately. The heat generated by the side reaction was obviously more abundant, and the flame size generated by combustion is larger, and all of them go through the stable combustion and mechanical rupture stage, which indicates that NCM523 battery store more energy, but the thermal safety is worse correspondingly.

### 2.2 Model Verification

**Figure 4:**
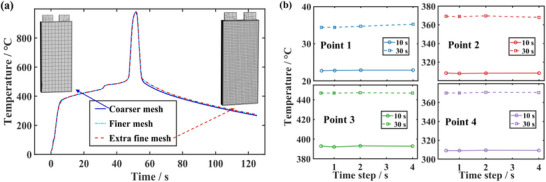
Verification of numerical discretization independence. a) Mesh independence verification. b) Time step sensitivity analysis.

To ensure the reliability of the simulation results, a mesh independence study and a time‐step sensitivity analysis are conducted. Successively finer mesh sizes and smaller time steps are tested until the difference in the predicted temperature is less than 2%. The final mesh configuration and time‐step settings, which meet this convergence criterion, are subsequently adopted for all models to guarantee both accuracy and computational efficiency. The independence of the mesh and time step verification results is shown in **Figure** **4**.

The deviation at the center point for both the finer and extra fine mesh remains within 2% at all times (Figure 4a). We selected four points on the front, interior, and back surfaces to investigate time step sensitivity, and collected temperature data at two moments. During non‐TR periods, step sizes of 0.5, 1, 2, and 4 s are selected. When time step down from 4 to 0.5 s, the maximum temperature deviation at the selected points is 2.5%. When stepping down from 2 to 0.5 s, the temperature deviation at all selected points remained below 1% (Figure 4b).

In this study, four key parameters are extracted from the temperature curves of NCM523 battery, which are the onset temperature of self‐heating (*T*_1_), the ignition time (*t*_1_), the ignition temperature (*T*_2_), and the maximum temperature of the battery surface (*T*_3_). Self‐heating begins with the decomposition of the SEI and can be directly captured through simulation. Ignition under extreme high‐temperature shocks corresponds to large‐scale TR inside the battery and is easily observable in experiments. Since the transition from self‐heating to ignition in LFP batteries occurs within 1 s, only three of the four characteristic parameters, *T*_2_, *T*_3_ and *t*_1_, are extracted for LFP batteries. Four parameters are defined as listed in **Table** **1**. For NCM523 battery, the high temperature rise rate before 10 s is entirely caused by the external high‐temperature. Because it is irrelevant to the TR of the batteries, the four characteristic parameters mentioned above are extracted from the data of the temperature curves after 10 s, similarly for LFP battery, the characteristic parameters are extracted from the data after 8 s.

**Table 1:** Definition of characteristic parameters.^[^ ^45^ ^]^

Characteristic parameters	Definition	Threshold
***T***_1_	The onset temperature of self‐heating	Temperature rise rate exceeds 8 °C s^−1^
***T***_2_	The onset temperature of ignition	Temperature rise rate exceeds 30 °C s^−1^
***T***_3_	The maximum temperature of battery surface	Peak of temperature curves
***t***_1_	The onset moment of ignition	The moment corresponding to *T*_2_

**Figure 5:**
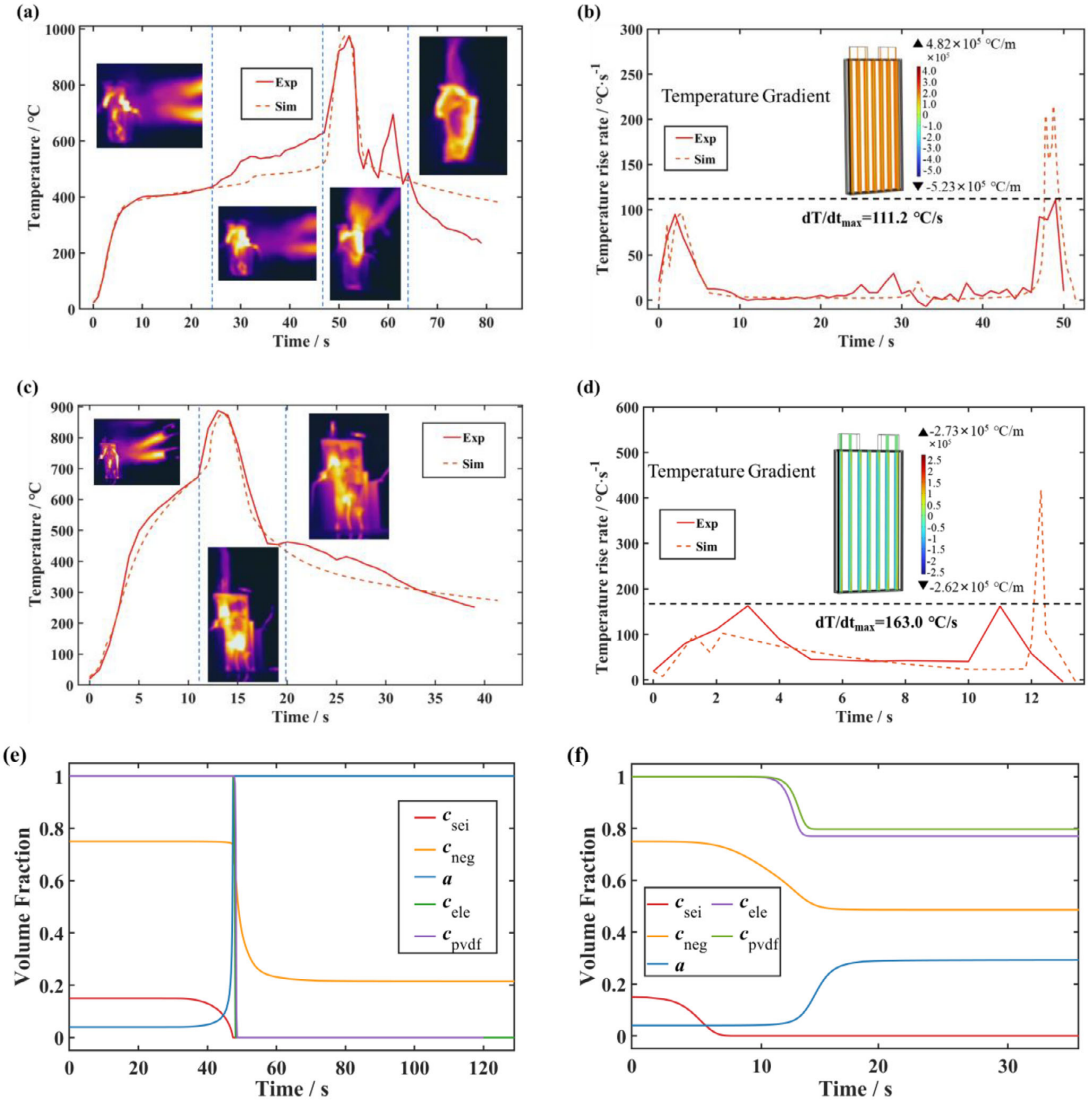
Model validation. a) The temperature versus time profiles of NCM523 battery. b) The temperature rise rate versus time profiles of NCM523 battery. c) The temperature versus time profiles of LFP battery. d) The temperature rise rate versus time profiles of LFP battery. e) Reactant conversion rates during TR of NCM523 battery. f) Reactant conversion rates during TR of LFP battery.

The experimental and simulation results for the two types of batteries are shown in **Figure** **5**. For NCM523 batteries, the simulation results are more accurate in stage I and stage III. Compared with the experimental results, the simulation results have some errors when TR occurs locally in stage II. This deviation may be because TR began in cores near the front before full combustion, which is also challenging to accurately model in terms of layer stratification. The simulation results are evaluated using the mean absolute percentage error (MAPE) and root mean square error (RMSE), which are calculated by Equations (1) and (2).

(1) $$MAPE=\frac{100\%}{n}\sum_{i=1}^{n}\left|\frac{{\hat{y}}_{i}-y_{i}}{y_{i}}\right|$$

(2) $$RMSE=\sqrt{\frac{1}{n}\sum_{i=1}^{n}{\left({\hat{y}}_{i}-y_{i}\right)}^{2}}$$

For the NCM523 battery, *MAPEs* between the experimental and simulation results are 7.06% for the first 52 s and 7.27% for the first 60 s, the *RMSEs* are 53.54 °C for the first 52 s and 54.70 °C for the first 60 s. For the LFP battery, the *MAPEs* and *RMSEs* are calculated to be 6.48%, 39.97 °C for the first 18 s, and 7.91%, 39.48 °C for the first 41 s. The prediction errors of the model for the key parameters are listed in **Tables** **2** and **3**. It can be seen that the model is accurate for the maximum surface temperature and ignition time, but the simulation of local TR is difficult to achieve. Figure 5e,f illustrates active material conversion. The chemical evolution follows the mechanism of TR. Changes in volume fraction partially confirm that LFP batteries undergo incomplete TR, where chemical substances are not fully converted. The validated model can be further used to 1) explore the connection between the surface temperature of the battery and the TR behavior; 2) estimate the TR behavior under the different SOCs; and 3) evaluate the TR behaviors at different distances from battery to external heat source. The strong agreement validates the model for the tested NCM523 and LFP pouch batteries. Its application to other chemical systems, forms, or operating conditions requires re‐parameterization and boundary condition settings based on their specific properties.

**Table 2:** Error of NCM523 battery model.

Characteristic parameters	Experimental values	Model values	TR model prediction errors
***T***_1_	435.6 °C	453.1 °C	4.02%
***T***_2_	630.1 °C	526.4 °C	16.5%
***T***_3_	973.6 °C	980.1 °C	0.68%
***t***_1_	47.0s	47.1s	0.21%

**Table 3:** Error of LFP battery model.

Characteristic parameters	Experimental values	Model values	TR model prediction errors
***T***_2_	650.4 °C	669.2 °C	2.89%
***T***_3_	888.0 °C	876.5 °C	1.30%
***t***_1_	10.2s	10.7s	4.90%

Notably, a relatively large deviation was observed between the simulated and experimental *T_2_* for the NCM523 battery. This is likely attributable to the immense challenge of precisely capturing the exact instant of ignition under such extreme, rapid‐transient heating conditions. The process is highly sensitive to local heterogeneities and thus also subject to experimental accidental errors. However, it is crucial to emphasize that this represents a localized temporal discrepancy rather than a systemic failure in the model's energy balance. The exceptional agreement in the predicted *T*_3_ and the overall temperature evolution confirms that the model accurately captures the essential energy dynamics of the event. Consequently, this deviation in *T*_2_ does not compromise the performance of the data‐driven models trained on the complete temperature profiles.

### 2.3 Estimation of Thermal Runaway Behaviors Under Different SOCs

**Figure 6:**
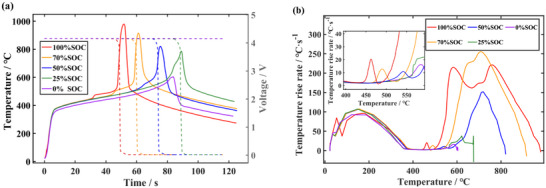
Comparison of different SOCs. a) The temperature and voltage versus time curves. b) The temperature rise rate versus temperature curves.

During the TR processes of LIBs, a significant portion of the heat is generated by electrochemical reactions of the energy stored in the batteries. The higher SOCs reduce the thermal stability owing to the increasing in the reactivity of the electrode materials. To accurately analyze the TR behavior of the battery, the effects of proposed model are verified under the different SOCs and the TR hazards of the batteries are explored. Meanwhile, the coupling relationship between four key parameters and SOCs can be analyzed. Five SOCs are selected, the surface temperature and the temperature rise rate curves of the batteries at 100% SOC, 70% SOC, 50% SOC, 25% SOC, and 0% SOC are shown in **Figure** **6**.

As shown in Figure 6a, decreasing SOC requires higher surface temperatures to ignite the internal cells, which indicating longer high‐temperature shock exposure. The heat released by the decomposition reaction also gradually decreases from stage II to VI. To trigger the battery TR, the continuous high‐temperature shock is employed until the batteries catch fire in tests and simulations. Notably, battery voltage will drop instantly in the ignition stage and mechanical damage occurs rapidly, particularly under higher SOCs.

**Figure 7:**
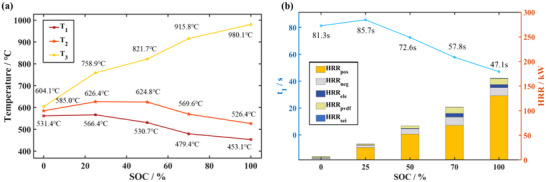
Variation of four characteristic parameters. a) *T*_1_, *T*_2_and *T*_3_ under different SOCs. b) *t*_1_ and peak HRR under different SOCs.

The changes of *T*_1_ and *T*_2_ of the battery under the different SOCs are shown in **Figure** **7**a. When the heat generated by decomposition of internal material is available to cause widespread TR, the SOCs decreasing will require higher temperatures for triggering battery TR. For the battery under 0% SOC, the maximum temperature rise rate is only 16.28 °C s^−1^ after 10 s, which is below the temperature rise rate during the fire started in the test. Therefore, for battery under 0% SOC, the temperature corresponding to the maximum temperature rise rate is regarded as *T*_2_. Figure 7a demonstrates the relationship between the characteristic temperature parameters and SOCs. The SOCs directly influences the energy stored and the thermal stability of the electrode material, thus the battery with higher SOCs release more heat and exhibit higher *T*_3_. It can also be seen that battery TR is less likely to be triggered in the lower SOCs range. Therefore, it requires longer time to heat for rising the internal temperatures.

The HRR is a major indicator for evaluating the thermal hazard of LIBs fires and is of great significance for the design of fire protection and fire suppression strategies for new energy vehicles. The bar graph in Figure 7b shows the peak HRR calculated in the simulation. Due to the high temperature of the external heat source shock, the TR occurs drastically with a high peak HRR. As the SOC decreases, the cathode decomposition heat release is reduced and the temperature rises slower. Thus, the peak HRRs of the TR side reactions also decrease, the peak HRR under 100%, 70%, 50%, 25%, 0% SOC are 165.3, 108.9, 69.1, 32.2, and 7.0 kW, respectively. It is shown that the SOCs has a significant effect on the HRR rate of chemical energy.

### 2.4 Estimation of Thermal Runaway Behavior at Different Flame Distances

**Figure 8:**
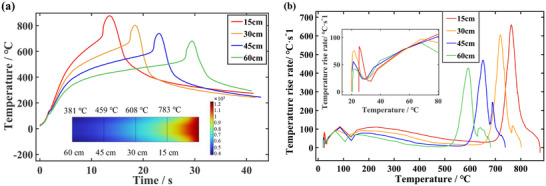
Comparison of different distance. a) The temperature versus time curves. b) The temperature rise rate versus temperature curves.

To analyze the distance from the heat source on the TR, the surface temperature and temperature rise rate curves at different distances are compared in **Figure** **8**. The distances between the external heat source and the battery surface directly affects the amount of heat received by the battery surface and efficiency of heat reception. In this section, based on the validated model of LFP batteries, the TR behaviors are predicted under the different distances from the heat source including 15, 30, 45, and 60 cm. In the subsequent Section 2.5, simulations are conducted with a distance of 75 cm to explore the reliability boundaries of the model.

The core objective of our experimental and simulation design to establish a series of well‐defined and reproducible thermal boundary conditions, rather than altering the “distance” itself. These conditions can induce TR within measurable and practical timeframes. The 15 cm distance is chosen to prevent direct flame impact on the battery surface. Flame impact causes localized intense heating and surface physical erosion, introducing complex factors difficult to model accurately. At distances much greater than 75 cm, the rate of heat dissipation to the surrounding environment approaches the rate of heat input, resulting in extremely slow temperature rise. This may prevent the battery from reaching the TR initiation temperature within a reasonable experimental timeframe. Certainly, 75 cm is not an upper limit but rather a prudent distance for reliability studies. Increasing the distance further, even if it still triggers thermal runaway, may fail to meet the research objective of simulating an “extreme high‐temperature impact” scenario. The subplot in Figure 7a displays a contour plot indicating the steady‐state temperature reaching the battery surface at different heat source distances. This quantitatively validates the distance selection rationale.

**Figure 9:**
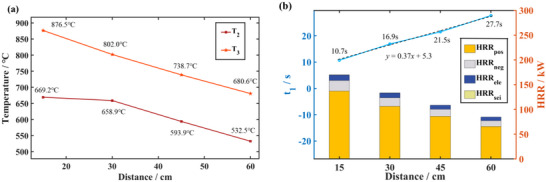
Variation of three characteristic parameters. a) *T*_2_ and *T*_3_ at different distances. b) *t*_1_ and peak HRR at different distances.

Since the tests are carried out in diabatic conditions, the convective heat transfer between the high‐temperature gas and the ambient air cannot be ignored. Therefore, distance greatly affects the heat exchange between the heat source and the environment. *T*_1_ of the LFP was difficult to extracted, because no abnormal increase in temperature is observed prior to ignition. **Figure** **9**a shows the relationship between characteristic temperatures and distances. As the heat is transferred from the aluminum‐plastic film shell to the internal cell, the heat source is still heating the shell, in addition, the heat input for the aluminium‐plastic film is larger than the heat output. Therefore, although the internal temperature changes little when ignition occurs at different distances, there is a significant difference in surface temperature. This phenomenon is more obvious in the diabatic heating condition. Considering the batteries have the same SOC and contain the same chemical energy inside, the temperature rises of the TR are also basically consistency. Thus, the *T*_3_ basically depends on *T*_2_ and the two curves are roughly parallel (Figure 9a). Figure 9b demonstrates the delay in ignition with the increase of distance from the heat source. With the heat source closer, the time for heat transfer to the battery surface is certainly shorter, thus the temperature rate of the battery surface rises faster. Importantly, once the high‐temperature heat source is located away from the battery surface, a large amount of heat dissipated through the heat convection between the external heat source and the ambient air. Therefore, the temperature is lower when it reaches the surface of the battery. The actual heat exchanged with the ambient air is greater than the simulated value because the high‐temperature gas affects the flow rate. Therefore, the drop in *T*_2_, *T*_3_ and the delay in *t*_1_ are more noticeable in real scenarios. In the range of 15–60 cm, the distance and *t*_1_ have a strong linear relationship, using the primary function *y* = 0.37*x* + 5.3 can be well fitted, where *x* denotes distance and *y* denotes *t*_1_, (Figure 9b, dotted line).

Distances directly affect the temperature of the heat source that reaches the surface of the battery; the closer the distance the higher the temperature. However, the chemical energy contained in the internal materials and the stored electrical energy differ little, thus HRR of each side reactions of TR decreases slightly as the distance gets further away. The peak HRR at 15, 30, 45, 60 cm are 169.8, 133.4, 108.6, and 84.8 kW, respectively. The results indicate that the distance from the heat source has a certain effect on the peak HRR, the internal temperature will be lower at a long distance when the chemical energy contained is basically same.

This study employed a targeted approach in modeling the two battery chemistries. For the NCM523 battery, the SOC factor is analyzed because its TR behavior is highly sensitive to SOC. For the LFP battery, the focus is placed on quantifying its response to varying thermal attack intensity (distance). This focus is chosen because its inherent thermal stability minimizes the sensitivity of its TR response to SOC changes. This implies that TR temperature curves at different SOC levels exhibit low distinguishability, potentially making it challenging for models to learn meaningful, generalizable mapping relationships in subsequent data‐driven analyses. Future work will aim to build a more comprehensive dataset that includes SOC effects for stable chemistries like LFP, further enhancing the generality of the proposed safety prediction framework.

### 2.5 Data‐Driven Model for Temperature Prediction with Real and Virtual Data

To improve the computational efficiency and reduce experimental cost, the data‐driven methods are employed to establish surrogate models using the real and virtual data for predicting the trends of TR temperatures. The virtual data are primarily collected from the multi‐physics TR model for compensating for the insufficient experimental data under the high‐temperature shock conditions. The surrogate models exploit the potential relationships from the historical or real‐time data through self‐learning, which avoids the necessity to study the complex electrochemical processes inside the LIBs. The latent information of the data and the connection between the TR temperature features are explored, and the convolutional combined long and short‐term memory networks based on the attention mechanism (CNN‐LSTM‐ATTENTION) are used to predict the TR surface temperature. Specifically, the training set comprises complete temperature‐time sequences from experiments and simulations at 15, 30, and 45 cm. The test set consists of the entire unseen temperature curve from the 60 cm simulation. Extrapolation to 75 cm further tests the limits of the model. Meanwhile, the data of different SOCs are obtained from the proposed model for predicting the temperature trends.

**Figure 10:**
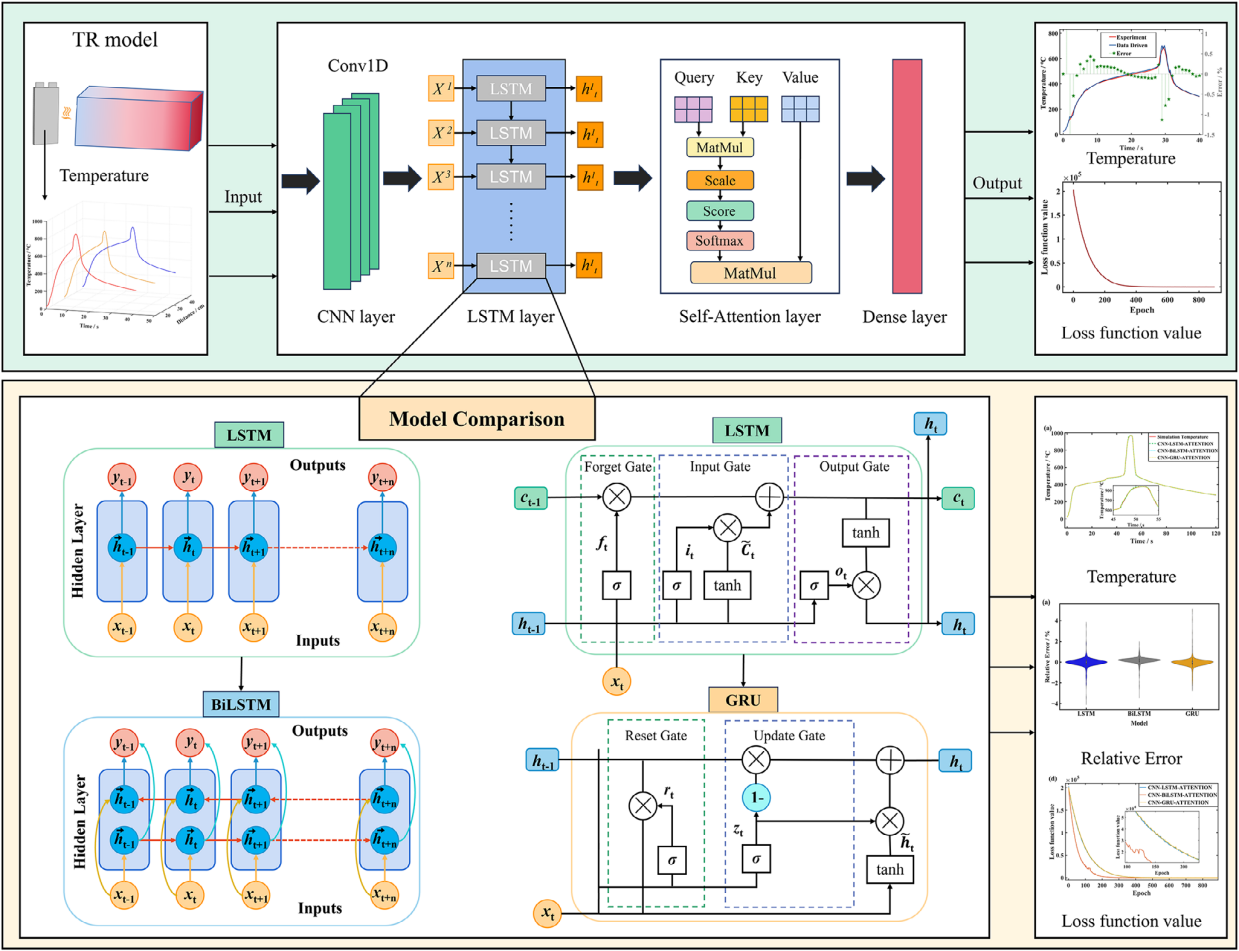
Schematic structure of CNN‐LSTM/BiLSTM/GRU‐ATTENTION module.

The schematic diagram of the neural network is shown in **Figure** **10**. The neural network architecture integrates sequential processing modules for temperature trend prediction. The input feature is historical time‐series data of the battery surface temperature. Scalar values as SOC or distance are not provided as additional input features, but are implicitly learned from the unique characteristics of the temperature curves. This is a common and effective practice when conditional variables profoundly influence the entire input sequence. Input features first pass through sequential convolutional operations with 3 × 3 kernels and padding of 1. The initial convolutional layer transforms single‐channel inputs into 16D feature representations, while the subsequent layer further processes these to 32D outputs. This progressive feature expansion enhances pattern recognition capability for detecting subtle thermal transients. This capability helps identify precursors to battery TR for providing earlier warning.

For learning the long‐term dependencies of time‐series temperature data, the three LSTM layer with the gate mechanism is employed to capture the flow of temperature information. The input data of the LSTM layer are obtained from the CNN layer with 32D features. The self‐attention layer is used to weight the output of the LSTM layer, converting the weights to probability distributions via the softmax function that assigns an attention weight to each LSTM output, thus allowing the model to focus on critical temperature change points. Finally, the context vector is mapped to the final output through the fully connected layer for realizing the temperature prediction. Owing to the traditional LSTM network drawbacks, the limited temperature data cannot be fully utilized and two typical advanced LSTM algorithm are applied. Compared with the traditional unidirectional LSTM, the BiLSTM neural network inherits the advantages of considering the influence of inputs before and after the current moment on the current output. Therefore, BiLSTM neural network can effectively improve the data utilization rate for improving the ability of the robustness and generalization for battery temperature prediction. Additionally, the GRU layer can be considered to replace the LSTM layer to achieve a faster running speed and expect to improve the model accuracy. All model variants share the same CNN frontend and self‐attention mechanism. To address overfitting, dropout combined with early stopping is employed. Dropout prevents the formation of complex co‐adaptive relationships between neurons, with a dropout rate set to 0.1. Early stopping terminates training prematurely by monitoring validation set error during training, halting when validation error begins to rise. Here, training stops if validation loss fails to improve over 50 consecutive epochs. Given the robust performance achieved using a standard set of hyperparameters (learning rate = 0.001, training epochs = 900, batch size = 32), and the high computational cost of tuning for each model variant, extensive automated hyperparameter search is not performed. All data‐driven models are encoded using PyTorch and trained on a computer with a 13th Intel(R) Core(TM) i9‐13900HX CPU 2.20 GHz and 16 GB of working memory (RAM). The models were solved and calculated using the GPU (NVIDIA GeForce RTX 4060 16 GB).

**Figure 11:**
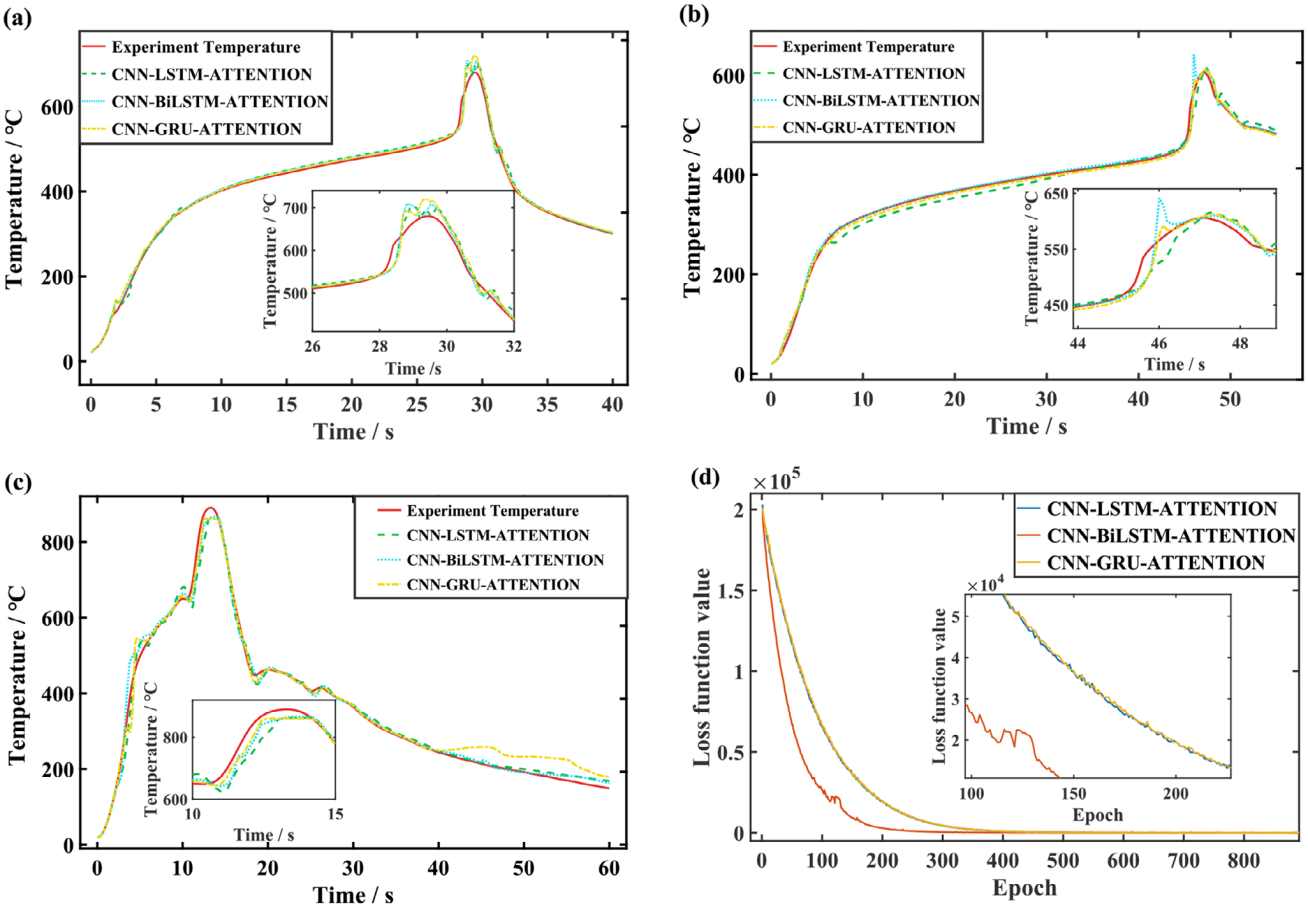
Comparison of temperature prediction results with simulation or test. a) Prediction of temperature at a distance of 60 cm from the heat source. b) Prediction of temperature at a distance of 75 cm from the heat source. c) Prediction of experimental temperature. d) Comparison loss function values for three prediction models.

The results of three predictions frameworks under different heat source distances are shown in **Figure** **11**a–c (Supporting Information). The temperature comparison of the TR part is shown in the sub‐figure zoomed in. Figure 11d shows the variation of loss function with iteration, the convergence speeds of the models with intermediate network as LSTM and GRU are close to each other, the loss function of the model with intermediate network as BiLSTM decreases significantly faster with the frequency of iteration. The data‐driven model predicts the future 0.5 s data using 1 s data, enabling a fairly accurate prediction of the time of TR occurrence and the maximum TR temperature.

**Figure 12:**
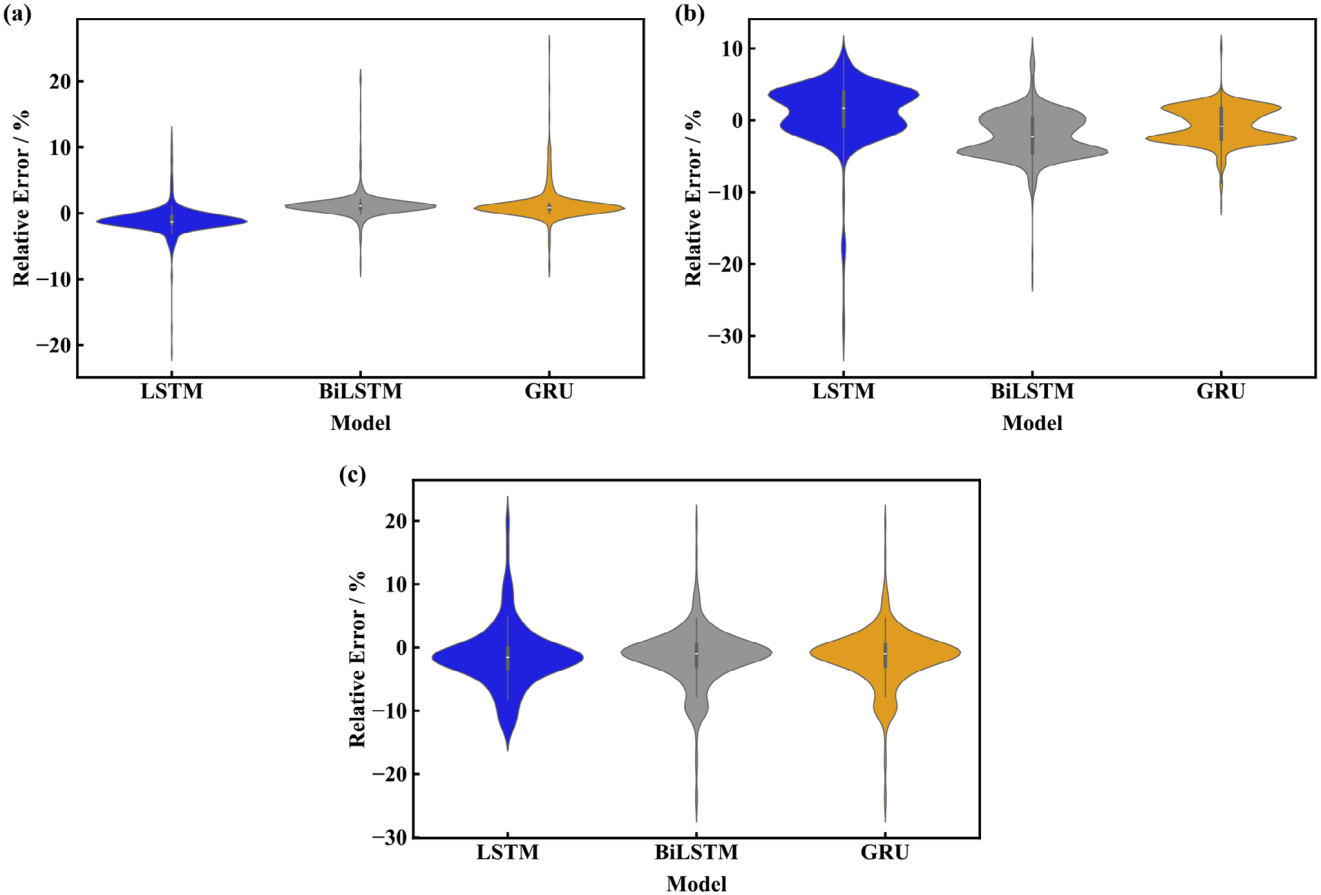
Relative errors of the three prediction frameworks. a) Prediction of the heat source distance of 60 cm. b) Prediction of the heat source distance of 60 cm. c) Prediction of experimental temperature.

The relative errors of prediction are shown in **Figure** **12**, the boxplot shows the temperature forecasting performance and distribution of the relative error, whereas the point marks indicate the relative prediction error including outlier. The upper and lower bounds of the relative errors of the three models in predicting the distance of 60 cm are −3.33% to 1.31%, −0.20% to 2.18%, and −0.15% to 1.76% respectively. The results of predicting a distance of 75 cm are shown in Figure 12b, the upper and lower bounds of the relative errors of the three models are −7.48% to 9.09%, −11.38% to 6.30%, and −8.62% to 4.53% respectively. Utilizing the above data in turn to predict the experimental data in order to further validate the validity of the model, the results of the prediction are shown in Figure 12c, the upper and lower bounds of the relative errors of the three models are −8.22% to 4.88%, −7.39% to 4.60%, and −32.25% to 24.02% respectively.

**Figure 13:**
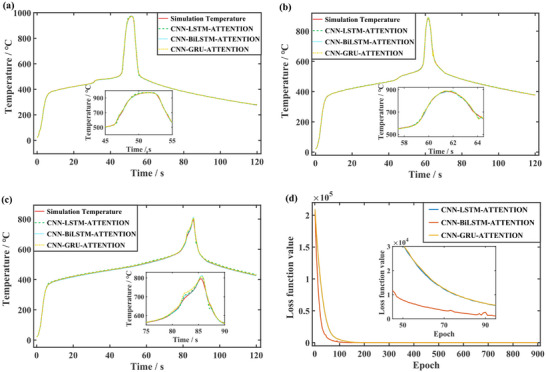
Comparison of temperature prediction results with simulation. a) Prediction of temperature under 90% SOC. b) Prediction of temperature under 66% SOC. c) Prediction of temperature under 33% SOC. d) Comparison loss function values for three prediction models.

For building the data‐driven models, the training datasets of the NCM523 battery are collected from the TR model under different SOCs including 100% SOC, 70% SOC, and 50% SOC. To verify the performances of the trained models, the models are utilized to predict the temperature profiles under 90% SOC, 66% SOC, and 33% SOC. The prediction results for different SOCs are shown in **Figure** **13**. The mean absolute errors (MAE) of the proposed models are kept within 0.5, 1.2, and 4.1 °C for the predicted three different SOCs, respectively. For the comparison of the three models, the maximum MAEs of the three predictions occur in the LSTM model, with the maximum MAEs of 21.20, 25.24, and 29.26 °C, besides the moments of the maximum MAEs are all during the TR. The model with BiLSTM as the intermediate framework still shows a significant advantage in convergence speed as shown in Figure 13d. The three frameworks decreased the loss function value by 99% at 128 iterations, 82 iterations, and 129 iterations, respectively.

**Figure 14:**
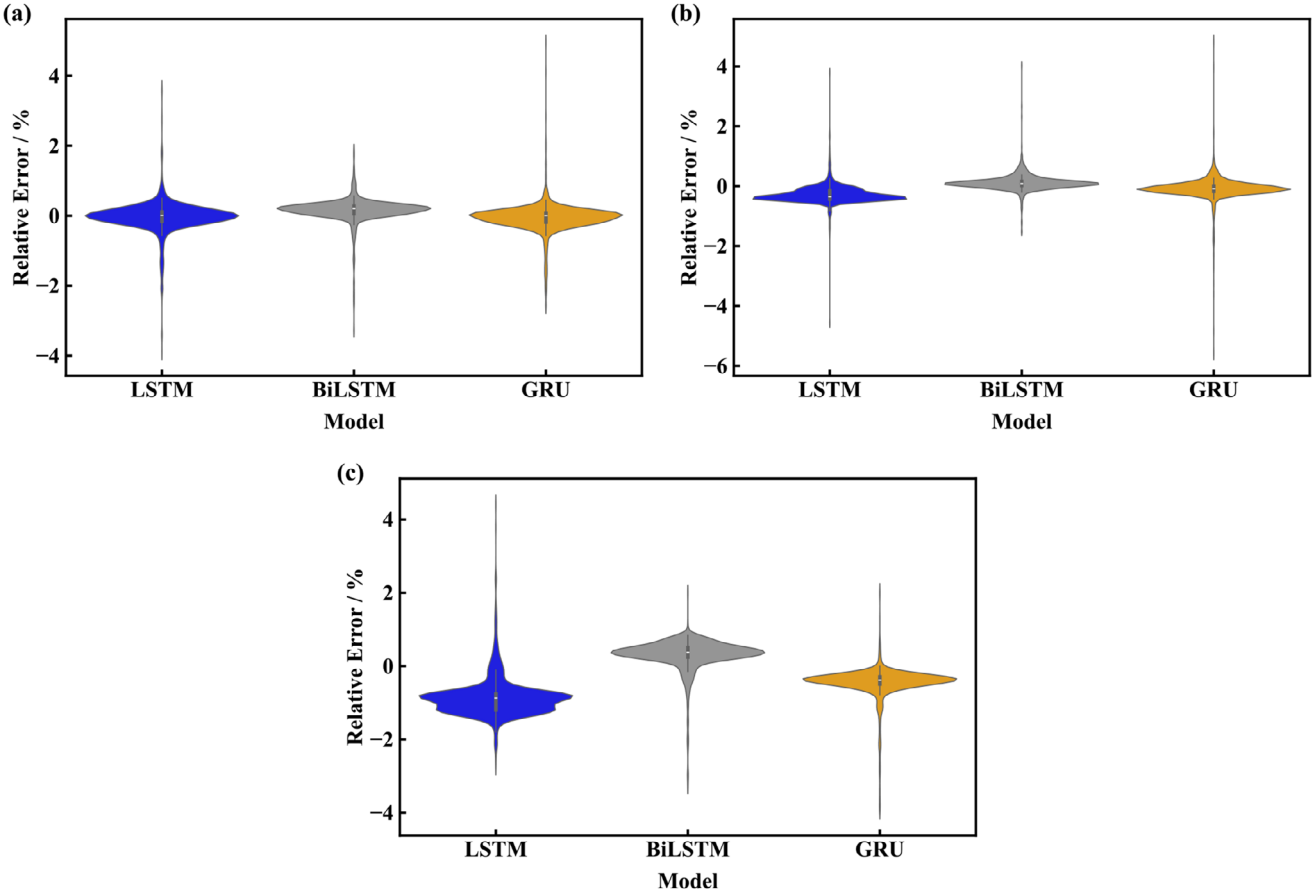
Relative errors of the three prediction frameworks. a) Prediction of 90% SOC. b) Prediction of 66% SOC. c) Prediction of 33% SOC.

The relative errors of prediction under 90% SOC are shown in **Figure** **14**a. The upper and lower bounds of the relative errors of the three models are −0.56% to 0.50%, −0.25% to 0.56%, and −0.54% to 0.45% respectively. The results of predicting 66% SOC and 33% SOC are shown in Figure 14b,c. The scopes of the relative errors of the three models in Figure 14b are −0.85% to 0.28%, −0.21% to 0.37%, and −0.42% to 0.26%, respectively. The corresponding data in Figure 14c are −1.80% to −0.09%, −0.15% to 0.83%, and −0.78% to 0.00% respectively. The results indicate the MAPE of the proposed models for all SOC predictions is less than 1% after removing outliers. The amount of data volume of training set will be larger when predicting conditions with lower external heat source temperatures and longer time to reach TR, thus the accuracy of the models is foreseen should still be maintained at this level.

Two criteria are incorporated to evaluate the prediction performance of the models from multiple perspectives: RMSE and MAPE. The evaluate results for the three scenarios for different distances are shown in **Table** **4**. Overall, the prediction accuracy is significantly decreased when predicting the experimental temperature due to the relatively small amount of data, whereas the prediction accuracy is improved when predicting 75 cm distance from the heat source with a high data volume. The model with the intermediate framework of BiLSTM showed the best prediction performance in all prediction scenarios. The model with the intermediate frame of GRU has only slightly worse accuracy than BiLSTM in predicting temperatures at heat source distances of 60 and 75 cm. Compared with other two models, the runtime efficiency of GRU is the highest with the prediction time of GRU at a distance of 60 cm being 70.49% of that of BiLSTM.

**Table 4:** Evaluation errors for the three cases.

Model	Criteria	60 cm from heat source	75 cm from heat source	Predict experiment	Runtime of 60 cm from heat source
LSTM	RMSE	9.03 °C	12.00 °C	18.76 °C	459.18 s
	MAPE	1.60%	3.17%	3.64%	
BiLSTM	RMSE	8.73 °C	6.74 °C	16.61 °C	561.08 s
	MAPE	1.43%	1.26%	3.02%	
GRU	RMSE	9.15 °C	6.75 °C	27.12 °C	395.48 s
	MAPE	1.49%	1.86%	8.02%	

With the larger amount of data are fed into the data‐driven models, all three frameworks show superior results when predicting temperatures at different SOCs than predicting different distances. Overall, the BiLSTM model showed the best accuracy followed by the GRU model, the RMSEs are below 4.2 °C and MAPEs are below 0.5% in all predictions. The LSTM model also kept the RMSE within 5 °C and MAPE within 1% for all three predictions. The model with the GRU intermediate framework still exhibits the fastest execution speeds, the running time is 88% of BiLSTM when predicting 33% SOC. The error results for the three scenarios under different SOC are listed in **Table** **5**.

**Table 5:** Evaluation errors for the three cases.

Model	Criteria	90% SOC	66% SOC	33% SOC	Runtime of 33% SOC
LSTM	RMSE	2.60 °C	1.97 °C	4.98 °C	665.99 s
	MAPE	0.25%	0.33%	0.93%	
BiLSTM	RMSE	2.22 °C	1.80 °C	2.93 °C	707.47 s
	MAPE	0.25%	0.17%	0.43%	
GRU	RMSE	2.08 °C	2.13 °C	4.11 °C	628.90 s
	MAPE	0.23%	0.21%	0.48%	

The model dataset enables robust predictions for the studied LIBs, demonstrating its strong ability to learn the patterns of TR evolution. A promising pathway for extending this framework is to utilize established modeling methods to generate virtual data for new systems. The key lies in embedding physical information constraints and battery material property parameters into the neural network. This ensures predictions for different battery systems and abuse conditions adhere to fundamental principles, thereby enhancing the reliability of extrapolation under limited data conditions.

## 3 Conclusion

In this study, the TR behaviors of NCM523 and LFP batteries under high‐temperature shock are investigated and the TR temperatures batteries are predicted using multiphysics and data‐driven model. Unique empirical data were generated through rigorously controlled flame exposure tests conducted on NCM523 and LFP batteries, addressing critical knowledge gaps in battery safety under real‐world fire scenarios. Based on the test results, a 3D conjugate heat transfer and TR coupling model is developed. Five characteristic parameters for analyzing the TR process, *T*_1_, *T*_2_, *T*_3_, *t*_1_ and HRR, are extracted from the temperature profiles and simulations. With the proposed NCM523 and LFP model, the higher SOC and closer distance to the heat source are derived to result in more prone to TR, higher TR temperature, and HRR. An innovative framework is developed through the integration of physics‐based simulation and deep learning methods. Multiphysics models are used to generate supplementary virtual data, enabling CNN‐LSTM/BiLSTM/GRU‐ATTENTION neural networks to predict TR temperature evolution beyond experimental test conditions. The results show that the neural network of real data combined with virtual data has excellent performance in the framework of predicting TR temperature. The neural network with BiLSTM displays the optimal performance in all six sets of predictions, achieving a MAPE below 4% under studied conditions. While GRU shows the shortest runtime, its accuracy proved unstable.

A framework for predicting TR temperature in LIBs under extreme high‐temperature shock conditions is proposed using experimental and virtual data. These advancements establish a new methodology for rapid battery safety assessment where physical testing is prohibitively hazardous. Future research will extend this framework to broader battery chemistries and multidimensional failure modes, accelerating the development of next‐generation battery safety engineering.

## 4 Experimental Section

### Battery Selection

Nickel‐cobalt‐manganese oxide (NCM) and LFP cathodes dominate the LIBs market due to their respective advantages in high energy density and intrinsic thermal safety.^[^ ^46^ ^]^ Among them, mid‐nickel NCM, which combines energy density and safety, had become the mainstream of the NCM family.^[^ ^47^ ^]^ Therefore, LIBs with these two representative cathodes were selected for comparative analysis of TR under high‐temperature impact. In this study, the pouch batteries mainly used in electric vehicles were tested, which consist of the electrode materials, including NCM523/graphite and LFP/graphite, respectively. The nominal voltage of NCM523 and LFP was 3.6 and 3.2 V, and charging cut‐off voltage of them was 4.2 and 3.6 V. Before the experiments, all the battery samples were fully charged in the constant current mode. The mass, internal resistance, dimensional information, and capacity of the batteries are listed in **Table** **6**.

**Table 6:** The specific parameters of batteries.

Type	NCM523	LFP
Initial mass (g)	298.38	470.35
Internal resistance (**Ω**)	1	0.552
Size (mm)	163.11 × 72.03 × 10.36	177.8 × 85.00 × 14.93
Cut‐off voltage (V)	4.2	3.6
Nominal voltage (V)	3.6	3.2
Capacity (Ah)	21.82	22.06

### Experimental Platform

The platform consists of flame heaters, pouch batteries, sensors, a data logger, infrared camera, and a bracket (Figure 3). The tests, unlike the oven test, were conducted in an open field with great ventilation condition, therefore, smoke buildup or explosions from battery failure cause no hazards. In the tests, the batteries were placed on the stand at roughly the same height as the heat sources, and the batteries were held in place on both sides by homemade fixtures. To minimize interference during testing, two mica plate fixtures were relatively small and clamped to the middle and bottom of the battery. The high‐temperature heat source emitted by the flame guns was generated by hydrogen combustion, with the combustion flame temperature ranging from 1100 to 1400 °C. Prior to testing, the pressure of the hydrogen flow and the distance from the heat source were adjusted to ensure that the temperature reaching the battery surface remains stable. The high‐temperature shock source generated by the hydrogen flame gun creates a thermal‐impact composite stress to simulate extreme fire exposure scenarios, while four positioned guns ensure comprehensive coverage of the front of the battery. To precisely define the thermal boundary condition, a blank calibration of the shock temperature was carried out before the test, and the thermocouple was placed in the same position as the test battery. Similarly, the temperature of the gas reaching the surface of the battery was ensured by controlling the temperature of the heat source and the convective heat transfer coefficient. The temperature profiles are shown in Figure 3, which proves that the external heat load was stable both in test and simulation. Temperature sensors and voltage sensors were equipped on the battery surface. The thermocouples were Omega K‐type, with a maximum temperature resistance of 1260 °C. High‐temperature resistant tapes were used to secure them to the battery surface to prevent detachment, meeting the temperature measurement requirements of the experiments. Voltage sensor clamped on the battery positive and negative tabs was used to collect battery voltage changes. The sampling rate of the data logger was set as 1 Hz to collect temperature and voltage data. The whole processes were recorded by a high‐resolution video camera.

### Experimental Process Configurations

The tests were carried out on the platform, as shown in Figure 3a. The camera and data logger were activated in advance for collecting the related data. For NCM battery, four guns were placed on the front side of the battery, with an initial distance of 40 cm between the battery and the guns, and the length of hydrogen flame was ≈10 cm. For LFP battery, since the heat source was only 15 cm away from the battery, the flame can roughly cover the front of the battery by only turning on the two guns. The hydrogen cylinder valve was immediately closed upon visual observation of sustained ignition on the battery surface, with this moment defined as the ignition time and temperature. For instance, under 40 cm calibration conditions, the flame temperature reaching the battery surface was ≈600 °C, and the NCM523 battery ignites after ≈44 s of exposure. Under 15 cm conditions, the flame temperature reaching the battery surface was ≈780 °C, and the LFP battery ignites after 12 s. Subsequently, the flame was observed to rapidly spread to the back of the battery. The TR behavior continued to be recorded until the surface temperature of the battery cooled down to the ambient temperature and the test was completed.

Four key parameters were observed including the onset temperature of self‐heating (*T*_1_), fire onset time (*t*_1_), the onset temperature of fire (*T*_2_), and the maximum temperature on the surface of the battery (*T*_3_). The temperature rise rate of the whole process, including flame gun ignition, TR onset, apparent combustion, and gradual cooling, can also be calculated from the surface temperature profile. The front of the battery would gradually fall off during the combustion process. Since the thermocouples were attached to the battery surface, the sensors cooled down in advance.

### Model Structure—Model Geometry and Boundary Conditions

**Figure 15:**
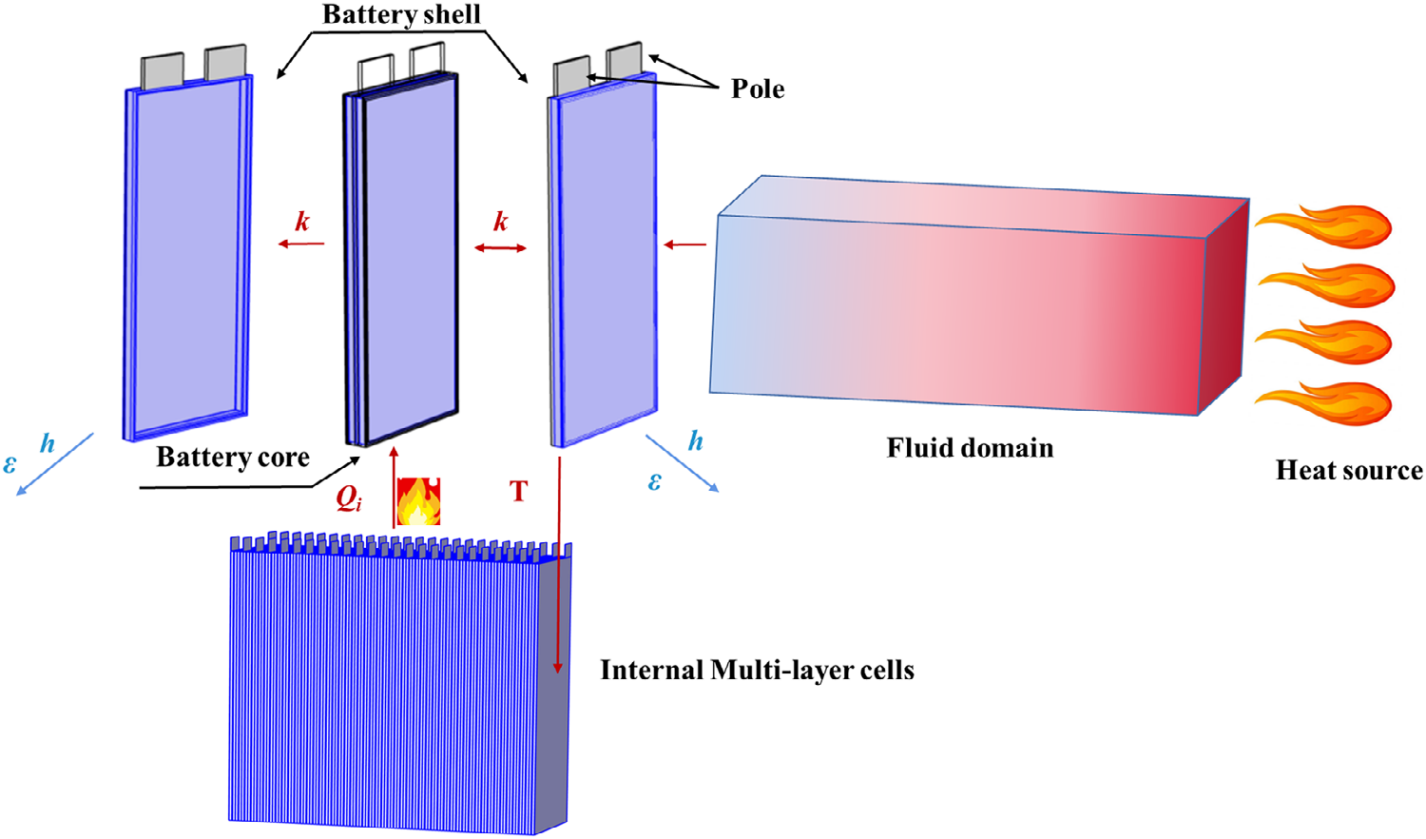
High‐temperature impact test geometry and heat transfer schematic.

The geometry of the battery consists of the battery shell, battery core, and two battery poles, with the external high‐temperature heating was represented using air columns. The single cell geometry was represented by positive collector, positive active layer, separator, negative active layer, negative collector, and poles as shown in **Figure** **15**. In this figure, *k* denotes the thermal conductivity, *h* denotes the convective heat transfer coefficient, ε denotes the thermal emissivity, *T* is the temperature of battery and ${\dot{Q}}_{i}$ is the internal thermal side reaction heat source.

The flame heating was simplified as a high‐temperature airflow boundary condition. This approach, while omitting the explicit modeling of radiative transfer and turbulence, was strategically chosen to enhance computational efficiency for the multi‐physics TR simulations. The convective heat transfer coefficient, as a critical boundary parameter, was not arbitrarily assumed but was rigorously derived through an inversion method. Specifically, the coefficient value was calibrated within a reasonable range, ensuring that the simulated temperature on the battery surface matches the experimental values obtained under no‐load conditions at different distances. This approach effectively represents the aggregate heat flux from the flame, and guarantees the accurate capture of the thermal load on the battery. While this simplification may not fully resolve the local fluid dynamics, its primary impact was on the precise path of heat delivery rather than the overall thermal energy received by the battery. Therefore, the methodology was confident that it had no materially undermine the accuracy of the subsequent TR simulations or the data‐driven models trained on their results.

Record the flame termination observed in the experiment, i.e., the moment when the battery ignites (*t*_1_). In the simulation, fluid inlet temperature was controlled via step functions to regulate the duration of high‐temperature shock. Ignition corresponds to large‐scale electrode decomposition within the main body of the core. At this time, the temperature at the fluid inlet instantly descend to ambient temperature.

### Model Structure—Heat Transfer Model in Solids and Fluids

In the proposed model, the general process of heat transfer occurs as high temperature of the flame reaches the front of the battery through the air fluid. The thermal conductivity of the aluminum‐plastic film was low, the heat accumulates rapidly in the shell, then conducted to the internal battery core. As the temperature rises, the internal core undergoes TR and generates a large amount of decomposition heat. At this time, the external heat source was turned off, the temperature of the battery continues to rise due to the heat generated by TR. After a rapid heating, battery temperature was much higher than the outside environment, thus thermal convection and thermal radiation occurring between the battery and the environment were considered. When the TR was finished, the temperature of the battery slowly drops to the ambient temperature.

For the proposed model, heat conduction consists of two parts including heat conduction in solid components and heat conduction defined in boundary conditions. Thermal conduction dominates the heat transfer within the battery, where an internal heat source exists can be described as Equation (3).

(3) $$\rho C_{p}\frac{\partial T}{\partial t}=\nabla \cdot \left(k\nabla T\right)+\sum {\dot{Q}}_{i}$$

where ρ denotes the density of the solid material, and *C_p_* is the constant pressure specific heat capacity.

The temperature and heat flux in this study were required to ensure their continuity at the interface between each calculation region. For the interface boundary conditions, Equations (4) and (5) illustrate the heat transfer at the interface between the battery shell and the internal core.

(4) $$T_{s,int}=T_{c,int}$$

(5) $$k_{s}{\left.\frac{\partial T_{s}}{\partial y}\right|}_{int,y=+0}=k_{c}{\left.\frac{\partial T_{s}}{\partial y}\right|}_{int,y=-0}$$

The interface between the fluid region and the lithium battery composite shell region belongs to the conjugate heat transfer. The heat transfer at the boundary satisfies Equations (6) and (7).

(6) $$T_{f,int}=T_{s,int}$$

(7) $$k_{f}{\left.\frac{\partial T_{f}}{\partial y}\right|}_{int,y=+0}+q_{r}=k_{c}{\left.\frac{\partial T_{s}}{\partial y}\right|}_{int,y=-0}$$

Heat transfer in high‐temperature fluids was dominated by thermal convection. When pressure work and viscous work were neglected, the fluid transient heat transfer was derived to account for the temperature field in the fluid, as in Equation (8).

(8) $$\rho C_{p}\frac{\partial T}{\partial t}+\rho C_{p}u\cdot \nabla T=\nabla \cdot \left(k\nabla T\right)+Q$$

Heat convection contains 1) high temperature gases in air and 2) the heat convection in the boundaries of solids and gases. There exists also thermal convection between a high‐temperature flame and the low‐temperature environment before it comes into contact with the battery. The boundary conditions for thermal convection can be described by Equation (9).

(9) $$q_{v}\left(T\right)=-k\frac{\partial T}{\partial n}=h\cdot \left(T_{0}-T_{\infty }\right)$$

where *q_v_* is the convective heat flux at the battery surface, *k* denotes the thermal conductivity of the solid component, $\frac{\partial T}{\partial n}$ denotes the temperature gradient at the boundary between the battery and the environment, *h* denotes the convective heat transfer coefficient between the battery surface and the environment, *T*_0_ is the temperature at the surface of the battery, and *T*_∞_ =  298.15 K (25 ^○^C) denotes the ambient temperature.

When TR occurs, the battery temperature was much higher than the ambient temperature, and the battery surfaces emit heat by radiating electromagnetic waves to the environment. The thermal radiation obeys the Stefan‐Boltzmann law and is represented by Equation (10), where *q_r_* is the radiant heat flux from the battery surface, and σ = 5.67 **×** 10^−8 ^W (m^2^·K^4^)^−1^ is the Boltzmann constant.

(10) $$q_{r}\left(T\right)=\varepsilon \sigma \left({T_{o}}^{4}-T_{\infty }^{4}\right)$$

During the heating phase, the intense convective heat input from the flame dominates by one to two orders of magnitude over radiative losses. Consequently, radiation exerts a negligible influence on predicting the onset time and peak temperature of thermal runaway. Moreover, accounting for thermal radiation from the heated surface at this stage further complicates the equivalence to convective processes. However, after flame turn‐off, radiation becomes a significant heat loss mechanism, and heat radiation from the heated surface returns to normal values (0.8 m^2^ s^−3^).

### Model Structure—Thermal Runaway Model

As the temperature of the battery rises, its internal materials begin to decompose. These exothermic decomposition reactions, referred to as side reactions, generate a large amount of heat in a short time, leading to TR. The side reactions mainly include the decomposition reaction of the SEI, the reaction of the negative electrode with the electrolyte, the reaction of the positive electrode with the electrolyte, and the decomposition reaction of the electrolyte and binder. The heat generated by the side reactions can be determined by Equations (11) and (12).

(11) $${\dot{Q}}_{i}=H_{i}\frac{d\alpha_{i}}{dt}$$

(12) $$H_{i}=q_{i}W$$

where *H_i_* is the enthalpy of a thermal reaction, α_*i*_ is the conversion fraction of reactant at each stage of the thermal reaction, *q_i_* is the exothermic heat of decomposition per unit mass of the material, and *W_i_* is the density of the material. Conversion rates at each stage of the reaction can be calculated by following the law of Arrhenius, Equation (13).

(13) $$\frac{d\alpha_{i}}{dt}=A_{i}\alpha_{i}\exp\left(-\frac{Ea_{i}}{RT}\right)$$

where *A_i_* is the frequency factor, *Ea_i_* is the activation energy; *R* is the ideal molar gas constant with the value of *R* = 8.314 J (mol·K)^−1^.

The decomposition of the SEI layer typically commences at ≈80 °C. The associated heat release is described by the following coupled Equations (14) and (15), which define the reaction rate and progress.

(14) $$Q_{\mathrm{sei}}=q_{\mathrm{sei}}W_{\mathrm{sei}}R_{\mathrm{sei}}$$

(15) $$R_{\mathrm{sei}}=-\frac{dc_{\mathrm{sei}}}{dt}=A_{\mathrm{sei}}c_{\mathrm{sei}}\exp\left(-\frac{Ea_{\mathrm{sei}}}{RT}\right)$$

Following the breakdown of the SEI layer, Li^+^ intercalated within the negative electrode graphite reacted directly with the electrolyte. This reaction is governed by Equations (16)–(18), where *z* is the dimensionless thickness of the SEI layer and *z*_0_ is its initial value.

(16) $$Q_{\mathrm{neg}}=q_{\mathrm{neg}}W_{\mathrm{neg}}R_{\mathrm{neg}}$$

(17) $$R_{neg}=-\frac{dc_{neg}}{dt}=A_{neg}c_{neg}\exp\left(-\frac{Ea_{neg}}{RT}\right)\exp\left(-\frac{z}{z_{o}}\right)$$

(18) $$\frac{dz}{dt}=A_{neg}c_{neg}\exp\left(-\frac{z}{z_{o}}\right)\exp\left(-\frac{Ea_{neg}}{RT}\right)$$

The decomposition of the cathode active material involves exothermic reactions with the electrolyte. Notably, the onset temperature and reaction kinetics differ significantly between cathode chemistries. For NCM523 cathodes, these reactions generally initiate upon exceeding 180 °C. For LFP cathodes, the onset occurs at a substantially higher temperature due to their superior thermal stability, typically above 240 °C.^[^ ^48^ ^]^ The heat generation is calculated by the following system of Equations (19) and (20), where *a* represents the conversion fraction of the cathode material.

(19) $$Q_{\mathrm{pos}}=q_{\mathrm{pos}}W_{\mathrm{pos}}R_{\mathrm{pos}}$$

(20) $$R_{\mathrm{pos}}=-\frac{da}{dt}=A_{\mathrm{pos}}a\left(1-a\right)\exp\left(-\frac{Ea_{\mathrm{pos}}}{RT}\right)$$

As the temperature continues to rise, the decomposition of the electrolyte and the PVDF binder occurs. The heat generated by these processes is described by Equations (21)–(24).

(21) $$Q_{\mathrm{ele}}=q_{\mathrm{ele}}W_{\mathrm{ele}}R_{\mathrm{ele}}$$

(22) $$R_{\mathrm{ele}}=-\frac{dc_{\mathrm{ele}}}{dt}=A_{\mathrm{ele}}c_{\mathrm{ele}}\exp\left(-\frac{Ea_{\mathrm{ele}}}{RT}\right)$$

(23) $$Q_{\mathrm{pvdf}}=q_{\mathrm{pvdf}}W_{\mathrm{pvdf}}R_{\mathrm{pvdf}}$$

(24) $$R_{\mathrm{pvdf}}=-\frac{dc_{\mathrm{pvdf}}}{dt}=A_{\mathrm{pvdf}}c_{\mathrm{pvdf}}\exp\left(-\frac{Ea_{\mathrm{pvdf}}}{RT}\right)$$

The primary control parameters for the TR side reaction are listed in **Table** **7**.

**Table 7:** Reaction kinetic parameters of internal LIB materials.^[^ ^15^, ^49^ ^]^

Electrode material	A_i_/s^−1^	Ea_i_/J·mol^−1^	H_i_/J·kg^−1^	W_i_/kg·m^−3^
SEI	1.67 × 10^15^	1.35 × 10^5^	2.57 × 10^5^	610
Graphite	2.5 × 10^13^	1.35 × 10^5^	1.71 × 10^6^	610
NCM523 (100%)	6.67 × 10^13^	1.24 × 10^5^	3.14 × 10^5^	122
NCM523 (70%)	1.28 × 10^11^	1.07 × 10^5^	3.14 × 10^5^	110
NCM523 (50%)	1.06 × 10^9^	9.25 × 10^4^	3.14 × 10^5^	76.7
NCM523 (25%)	8.04 × 10^9^	9.15 × 10^4^	3.14 × 10^5^	38.3
NCM523 (0%)	9.19 × 10^7^	7.42 × 10^4^	3.14 × 10^5^	0
LFP	1.74 × 10^9^	1.15 × 10^5^	1.03 × 10^5^	121
Electrolyte	2.5 × 10^13^	2.74 × 10^5^	1.55 × 10^5^	407
Binder	1.91 × 10^25^	2.86 × 10^5^	1.50 × 10^6^	81.4

### Model Structure—Estimation of Reaction Kinetics

Changes in the battery SOC determine the lithiation state of the negative electrode and the stored energy, significantly influencing the thermal stability of the active materials. The core was located inside the battery, before the shell ruptures, all the self‐generated heat was transferred to the battery itself, so the core was considered in an adiabatic environment, following Equation (15), where *V* denotes volume of reaction area. According to Equations (13) and (17), simplified to Equation (18).

(25) $$H=C_{p}\cdot V\cdot W_{i}\cdot \Delta T$$

(26) $$\ln\left(\frac{\frac{dT}{dt}}{T-T_{0}}\right)=\ln A_{i}-\frac{Ea_{i}}{R}\cdot \frac{1}{T}$$

where the reaction finger front factor *A_i_* and activation energy *Ea_i_* can be calculated from the intercept and slope, respectively, by a linear fit of $\ln(\frac{\frac{dT}{dt}}{T-T_{0}})$ to $-\frac{1}{T}$. However, due to the reliance on calorimetric test data, the above method can only model the TR behavior of LIBs at a specific SOC. Therefore, *A_i_* and *Ea_i_* can only be estimated from temperature intervals.

All the governing equations above were implemented in a finite element simulation. The multiphysics simulations were performed using the COMSOL Multiphysics 6.1 and employed the PARDISO solver to solve the linear equation system. The computational domain was discretized with a physics‐controlled mesh, featuring refined elements in critical regions (Supporting Information).

## Conflict of Interest

The authors declare no conflict of interest.

## Supporting information



Supplemental Data File 1

## Data Availability

The data that support the findings of this study are available from the corresponding author upon reasonable request.
